# Incidence and characteristics of death from peptic ulcer among cancer patients in the United States

**DOI:** 10.1038/s41598-021-00602-1

**Published:** 2021-12-08

**Authors:** Pengcheng Yang, Yongqiang Zheng, Lei Zhang, Xiaohua Hou

**Affiliations:** 1 Division of Gastroenterology, Union Hospital, Tongji Medical College, Huazhong University of Science and Technology, Jiefang Avenue 1277, Wuhan, 430022 China; 2 State Key Laboratory of Oncology in South China, Sun Yat-Sen University Cancer Center, Sun Yat-Sen University, Guangzhou, 510060 China

**Keywords:** Cancer epidemiology, Peptic ulcers, Risk factors

## Abstract

Most cancer patients die of non-cancer causes, and peptic ulcer is one cause that deserves attention. To characterize the incidence and risk factors of death from peptic ulcer among cancer patients, we extracted the data of cancer patients registered in the Surveillance Epidemiology and End Results (SEER) program from 1975 to 2016. Out of the 8,471,051 patients extracted from SEER, 4,698 died from peptic ulcer, with a mortality rate of 9.08/100,000 person-years. Meanwhile, the mortality rate in the general population was 5.09/100,000 person-years, giving a standardized mortality ratio (SMR) of 1.78 (95% confidence interval, 1.73–1.84). Patients who are female, of other race, unmarried, and with distant tumor stage have greater SMRs. A higher SMR was associated with a younger age at diagnosis. Among those aged < 40 years at diagnosis, the plurality of fatal peptic ulcers occurred in patients with leukemia and lymphoma, while in patients aged > 40 years, the majority occurred in those with prostate, breast, colorectum, and lung cancer. Patients with upper digestive system malignancies had the highest SMRs and hazard ratios (HRs), which could be ascribed to radiotherapy-induced damage to the gastroduodenum. The risk declined rapidly one year after diagnosis. However, the SMRs in the upper digestive system cancer survivors increased significantly over ten years after diagnosis. Upper digestive system cancers adjacent to the gastroduodenum were associated with higher SMRs and HRs compared with other types of cancer, possibly contributing partially to the damage caused by radiotherapy on the radiosensitive gastroduodenum.

## Introduction

Cancer is currently the second leading cause of death in the United States^1^. Peptic ulcer is a common chronic disease in modern society, given the increased pressure individuals face from work and life. Once perforation or vascular invasion occurs and causes massive bleeding, peptic ulcers can be fatal. Despite the technical progress in recent years, the mortality rate of acute peptic ulcer bleeding has remained at approximately 10%^2^. Cancer can lead to ulcers or aggravate existing ulcers via local tumor invasion^3^, vascular embolization^4^, radiation damage from radiotherapy^5–7^, gastroduodenal toxicity of chemotherapeutics^8,9^, shared lifestyles among cancer survivors, or other pathways unclear to date. Due to medical advancements, the number of cancer survivors has been increasing^10^. It is predicted that the number of Americans with a history of cancer will rise to over 26 million by 2040^11^. Hence, it is crucial to identify cancer patients with an increased mortality risk of peptic ulcers to prevent potential risks.

The National Comprehensive Cancer Network offers survivorship guidelines after cancer treatment to reduce long-term morbidity and mortality^12^. Until now, there has been relatively limited guidance in peptic ulcer prophylaxis, identification, or management, specifically for patients with cancer. Therefore, there are limited resources currently available to help clinicians, including oncologists, gastroenterologists, and care physicians, in identifying cancer survivors who are at an increased mortality risk of peptic ulcers. Characterizing and targeting subgroups at an elevated risk of fatal peptic ulcers among all patients can be an effective strategy to protect cancer survivors.

Our research aimed to characterize the patterns and risk factors of peptic ulcer mortality among cancer patients in the United States and identify the demographic and disease characteristics, such as tumor stage, that are associated with an elevated risk of peptic ulcer mortality. Our study might be clinically beneficial for physicians in charge of survivorship management to alleviate the mortality risk of peptic ulcers in cancer survivors and improve their long-term survival.

## Results

We identified 8,471,051 cancer patients in the Surveillance, Epidemiology, and End Results (SEER) program between 1975 and 2016. Among these patients, 4,698 died of peptic ulcer(s) (Table 1). The mortality rate was 9.08 per 100 000 person-years. In contrast, the corresponding rate of death from ulcers in the general US population was 5.09 per 100 000 person-years, revealing a standardized mortality ratio (SMR) of 1.78 (95% confidence interval [CI] 1.73–1.84).

**Table 1:** Incidence and SMRs of gastroduodenal ulcer mortality among cancer patients.

Characteristic	Cancer patients		Ulcers		Death rate^a^	SMR^ab^	95% CI
	No	%	No	%			
**Sex**							
Male	4,202,426	49.61	2629	55.96	10.45	1.53	1.44–1.62
Female	4,268,625	50.39	2069	44.04	7.92	2.03	1.89–2.19
**Race**							
White	7,036,840	83.07	3983	84.78	8.88	1.72	1.64–1.81
Black	861,518	10.17	386	8.22	9.70	1.96	1.65–2.32
Other	572,693	6.76	329	7.00	10.79	3.26	2.61–4.07
**Marital status**							
Married	4,679,040	55.24	2424	51.60	8.34	1.67	1.56–1.78
Unmarried	3,194,607	37.71	2013	42.85	11.21	2.07	1.91–2.23
Unknown	597,404	7.05	261	5.56	6.59	1.28	1.06–1.53
**Tumor stage**							
In situ	483,079	5.70	177	3.77	4.60	0.90	0.73–1.1
Localized	3,144,583	37.12	1841	39.19	6.66	1.31	1.22–1.4
Regional	1,391,229	16.42	869	18.50	11.12	2.19	1.94–2.46
Distant	1,463,749	17.28	582	12.39	17.96	3.40	2.87–4.03
Unstaged	1,988,411	23.47	1229	26.16	13.92	2.66	2.39–2.96
**Year of diagnosis**							
1975–1989	1,104,822	13.04	1653	35.19	19.73	3.97	3.57–4.42
1990–1999	1,319,905	15.58	1241	26.42	10.68	2.09	1.9–2.31
2000–2009	3,422,077	40.40	1366	29.08	5.63	1.10	1.02–1.19
2010–2016	2,624,247	30.98	438	9.32	5.94	1.14	0.99–1.3
**Surgery**							
Yes	5,067,632	59.82	2840	60.45	7.74	1.53	1.44–1.62
No	3,274,257	38.65	1787	38.04	13.13	2.37	2.18–2.58
Unknown	129,162	1.52	71	1.51	14.53	2.71	1.73–4.24
All cancer	8,471,051	100.00	4698	100.00	9.08	1.78	1.73–1.84
General population			257,327		5.09	Reference	

^a^Adjusted for age distribution of the cancer population in the SEER program.

^b^For the categories of sex and race, the reference population for SMR was the specific category in the subpopulation of general US people (e.g., the SMR for males is the observed number of deaths in men with cancer divided by the expected number of deaths based on the rate of men in the general population). For marital status, stage at presentation, surgery, chemotherapy, radiotherapy, and year of diagnosis, the reference population for SMR was the entire general US population from 1975 to 2016.

### Characteristics associated with a high risk of mortality

Peptic ulcer had a higher mortality rate among males, other race, and those who were unmarried (Table 1). Regarding the tumor stages, patients with metastatic/distant disease had the highest SMR for peptic ulcer death (3.40; 95% CI 2.87–4.03). Those diagnosed in 1975–1989 had an SMR of 3.97 (95% CI 3.57–4.42), and this gradually declined with time. Patients who had undergone surgery had a lower risk of death from peptic ulcers than those who did not. A higher SMR for peptic ulcer death was associated with a younger age at diagnosis, and the SMRs gradually decreased with increasing age at diagnosis (Table 2).

**Table 2:** Mortality rates of fatal gastroduodenal ulcer in persons with cancer by age at diagnosis.

Age at diagnosis (years)	No. of deaths from ulcers	Survival time (person-years)	Mortality rate of ulcer in cancer population^ab)^	Death rate of ulcer in general US population^a^	SMR^b^	95% CI	Death rate of ulcer in female cancer survivors^a^	Death rate of ulcer in male cancer survivors^a^
0	0	67,484	0.00	0.32	0.00	0–0	0.00	0.00
01–04	2	249,361	0.80	0.02	33.42	8.29–134.7	0.00	1.48
05–09	1	177,121	0.56	0.01	56.46	7.87–405.15	0.00	1.03
10–14	1	201,375	0.50	0.01	38.20	5.34–273.33	0.00	0.97
15–19	4	361,894	1.11	0.03	40.94	15.25–109.89	1.07	1.15
20–24	0	606,061	0.00	0.05	0.00	NA	0.00	0.00
25–29	5	978,734	0.51	0.09	5.55	2.31–13.38	0.00	1.28
30–34	7	1,438,160	0.49	0.16	3.12	1.48–6.56	0.43	0.59
35–39	27	2,065,911	1.31	0.29	4.52	3.1–6.61	0.91	2.18
40–44	55	3,095,861	1.78	0.52	3.40	2.6–4.43	0.90	3.98
45–49	74	4,250,741	1.74	0.91	1.91	1.52–2.4	1.37	2.57
50–54	211	5,459,542	3.86	1.42	2.72	2.37–3.11	2.80	5.52
55–59	305	6,383,648	4.78	2.27	2.11	1.88–2.36	3.96	5.70
60–64	453	6,844,210	6.62	3.47	1.91	1.74–2.1	6.18	7.02
65–69	672	6,817,277	9.86	5.20	1.90	1.76–2.05	8.89	10.64
70–74	813	5,585,578	14.56	8.00	1.82	1.7–1.95	12.91	15.88
75–79	822	3,928,983	20.92	12.48	1.68	1.56–1.8	18.75	22.92
80–84	657	2,125,066	30.92	19.72	1.57	1.45–1.69	26.23	36.26
85+	589	1,110,246	53.05	35.88	1.48	1.36–1.6	48.27	60.14

^a^Per 100,000 person-years.

^b^Reference population: general US population, 1969 to 2016.

### Tumor types associated with high mortality risks

**Figure 1:**
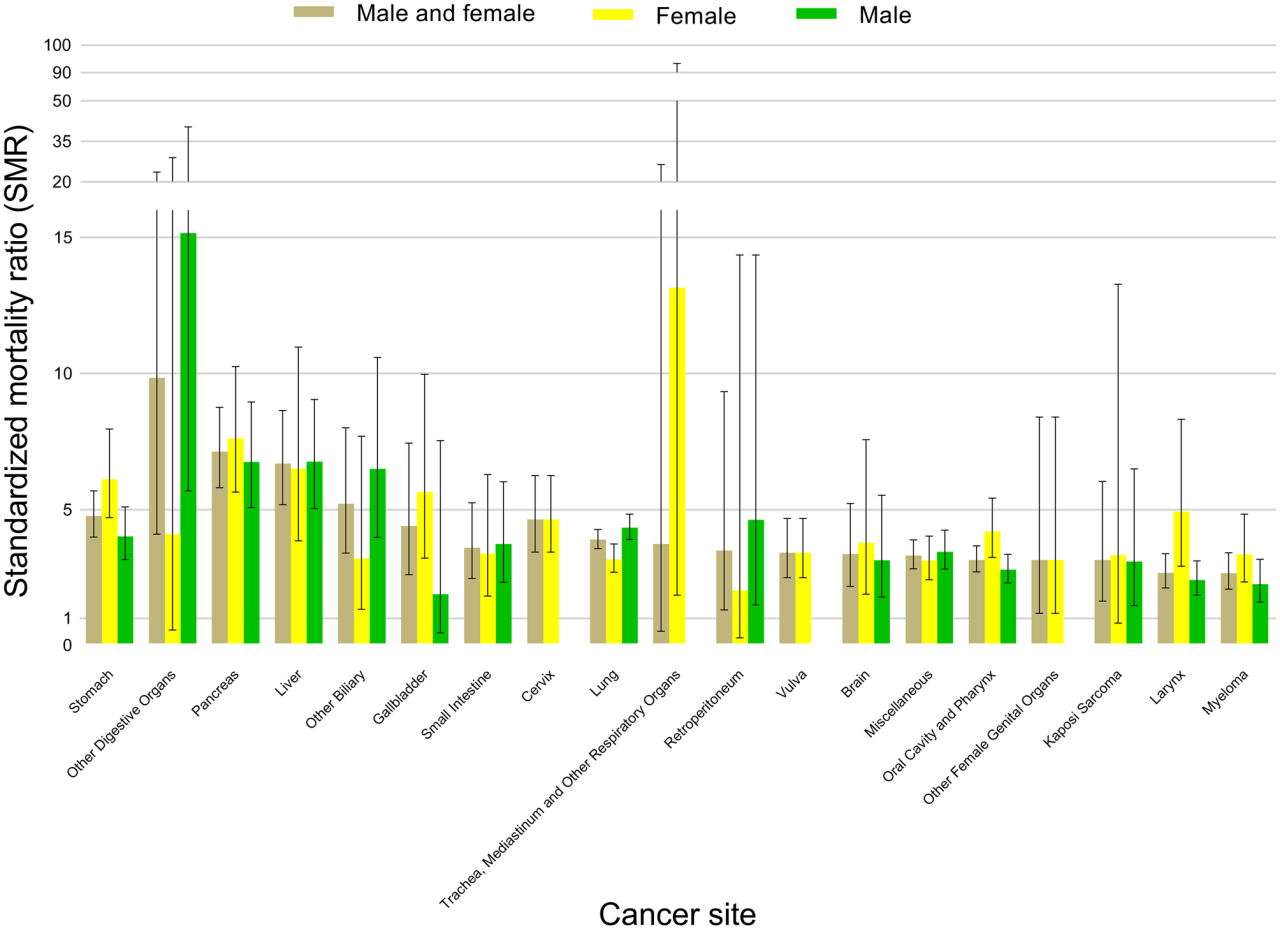
Standardized mortality ratios (SMRs) of fatal peptic ulcer among cancer patients by cancer site. These 19 cancer sites were chosen because they represent the sites with the highest SMRs. The y-axis depicts the SMR with a 95% confidence interval (CI), and the x-axis depicts the disease site. The sexes are shown in different colors. Cancers of the upper digestive system (stomach, small intestine, gallbladder, other biliary sites, liver, pancreas, and other digestive organs) have higher SMRs. Error bars represent the 95% CI by site.

**Figure 2:**
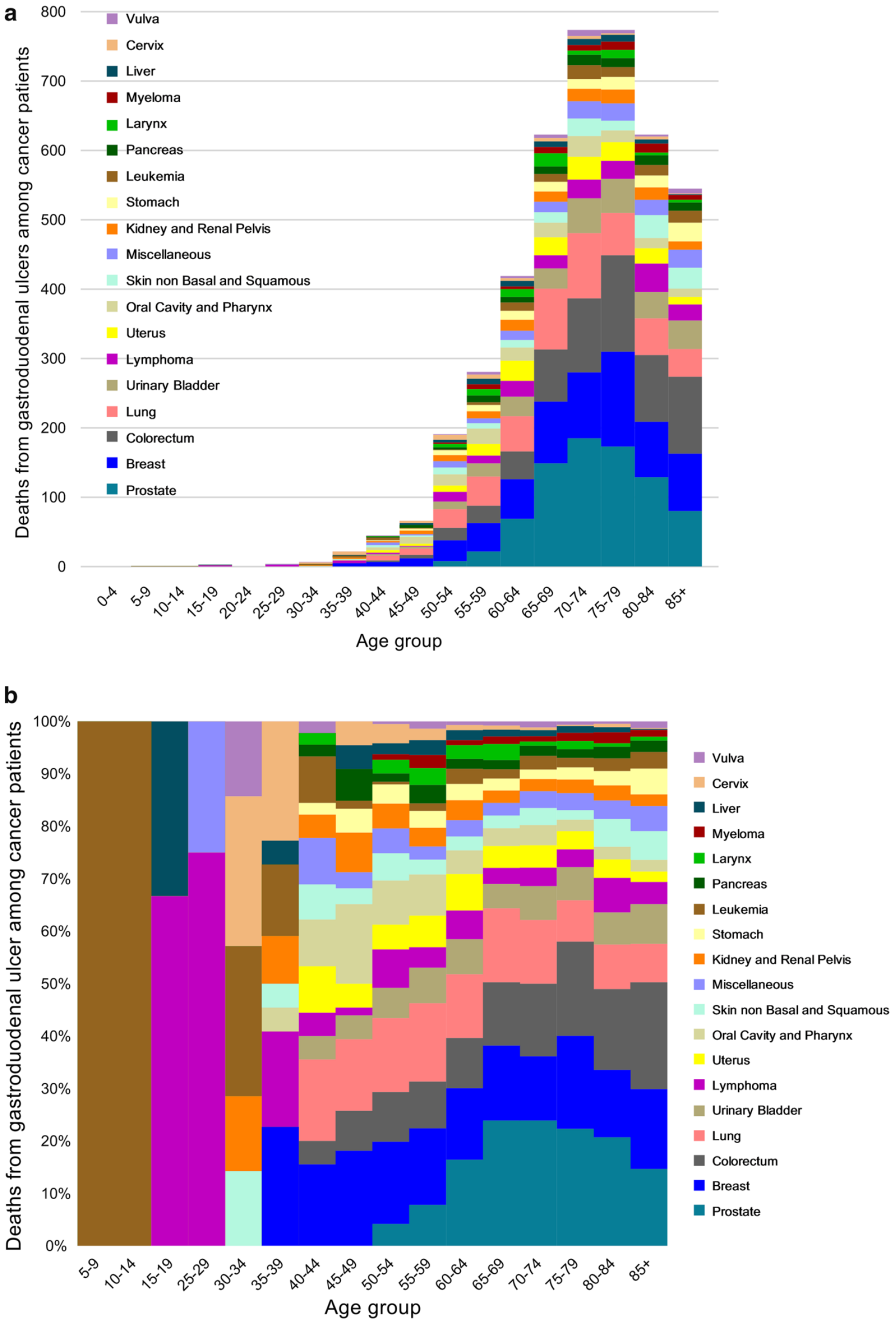
Fatal peptic ulcers among cancer patients as a function of cancer site and age at diagnosis. The colors depict the disease sites. (**a**) The y-axis depicts the absolute number of deaths from peptic ulcers, and the x-axis depicts the age group at the time of diagnosis. Most of the ulcer deaths occurred in patients diagnosed at an older age (i.e., ≥ 50 years) and those with prostate, breast, colorectum, and lung cancer. These 19 cancer sites were chosen because they represent the sites with the most deaths from peptic ulcers. (**b**) The y-axis depicts the relative number of fatal ulcers compared with all cancer patients, and the x-axis depicts the age group at the time of diagnosis. For children, adolescents, and young adults (i.e., < 40 years), the plurality of peptic ulcer deaths was observed in those with leukemia and lymphoma. By contrast, among older adults (i.e., > 40 years), the plurality of fatal ulcers occurred in those with prostate, breast, colorectum, and lung cancer.

Figure 1 and Supplementary Table 2 show the mortality rate and SMRs of peptic ulcer death among cancer patients by cancer site. Although the SMRs in patients were higher for most cancer types compared to the general US population, the mortality risks were greatest in survivors with malignancies of other digestive organs (ICD-10 code C26, including cancer of intestinal tract part unspecified, spleen nonlymphoma, overlapping lesion of the digestive system, and ill-defined sites within the digestive system; SMR = 9.84; 95% CI 4.09–23.64), followed by pancreatic cancer (ICD-10 code 25; SMR = 7.13; 95% CI 5.80–8.75), cancers of the liver (ICD-10 code C22; SMR = 6.69; 95% CI 5.18–8.63), cancers of other biliary sites (ICD-10 code C24, including cancer of extrahepatic bile duct, ampulla of Vater, overlapping lesion of the biliary tract, and biliary tract unspecified; SMR = 5.21; 95% CI 3.40–8.00), and stomach cancers (ICD-10 code C16; SMR = 4.76; 95% CI 3.99–5.68). Moreover, malignancies of the pancreas and liver remained related to high SMRs in cancer survivors of both sexes (Fig. 1). Patients with upper digestive system tumors adjacent to the gastroduodenum (including the esophagus, small intestine, pancreas, liver, gallbladder, other biliary sites, and other digestive organs) showed greater mortality risks of peptic ulcer than those with other cancers (Supplementary Table 2, Fig. 1). Figure 2 shows fatal ulcers among cancer patients as a function of age at diagnosis in different types of malignancies. Relatively few patients aged < 40 years died of peptic ulcers, partly because most cancers are diagnosed in the elderly. Among patients diagnosed at age < 40 years, the plurality of ulcers occurred in patients with leukemia and lymphoma. By comparison, in patients diagnosed at age > 40 years, the plurality of fatal ulcers occurred in patients with prostate, breast, colorectal, and lung cancer.

### Fatal peptic ulcer risk over time after diagnosis

**Figure 3:**
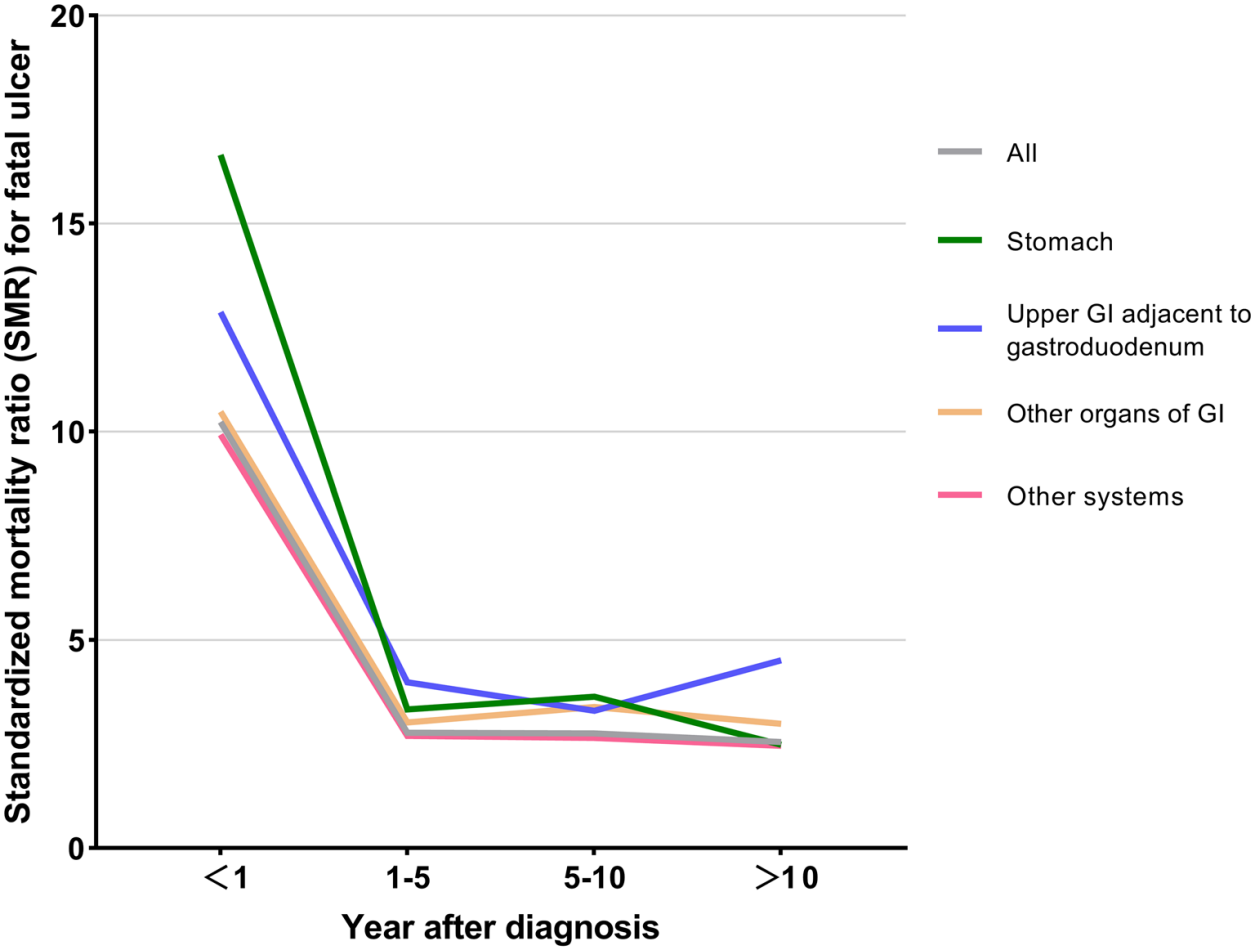
Standardized mortality ratios of fatal peptic ulcer were presented by cancer site and follow-up time. The upper gastrointestinal organs (GI) adjacent to gastroduodenum include the esophagus, small intestine, pancreas, liver, gallbladder, other biliary sites, and other digestive organs. The other organs of the GI include the retroperitoneum, colorectum, and anus. For most cancer types, the relative risk for fatal peptic ulcer was highest in the period immediately after cancer diagnosis, but this decreased rapidly thereafter. The SMRs for the upper digestive system malignancies adjacent gastroduodenum were significantly elevated 10 years after diagnosis, compared with that in other types of cancer.

Overall, the increased relative risk for peptic ulcer death among cancer patients was highest in the first year after diagnosis, which then markedly decreased thereafter. Despite the fluctuations, cancer patients (all anatomic sites) had elevated SMRs compared with the general population. At 10 years after diagnosis, the SMRs of fatal ulcers in malignancies of the upper digestive system adjacent to the gastroduodenum (including the esophagus, small intestine, pancreas, liver, gallbladder, other biliary sites, and other digestive organs) were relatively high compared with those of other cancers (Supplementary Table 1, Figure 3).

### Radiotherapy was associated with elevated mortality risk of peptic ulcer

**Figure 4:**
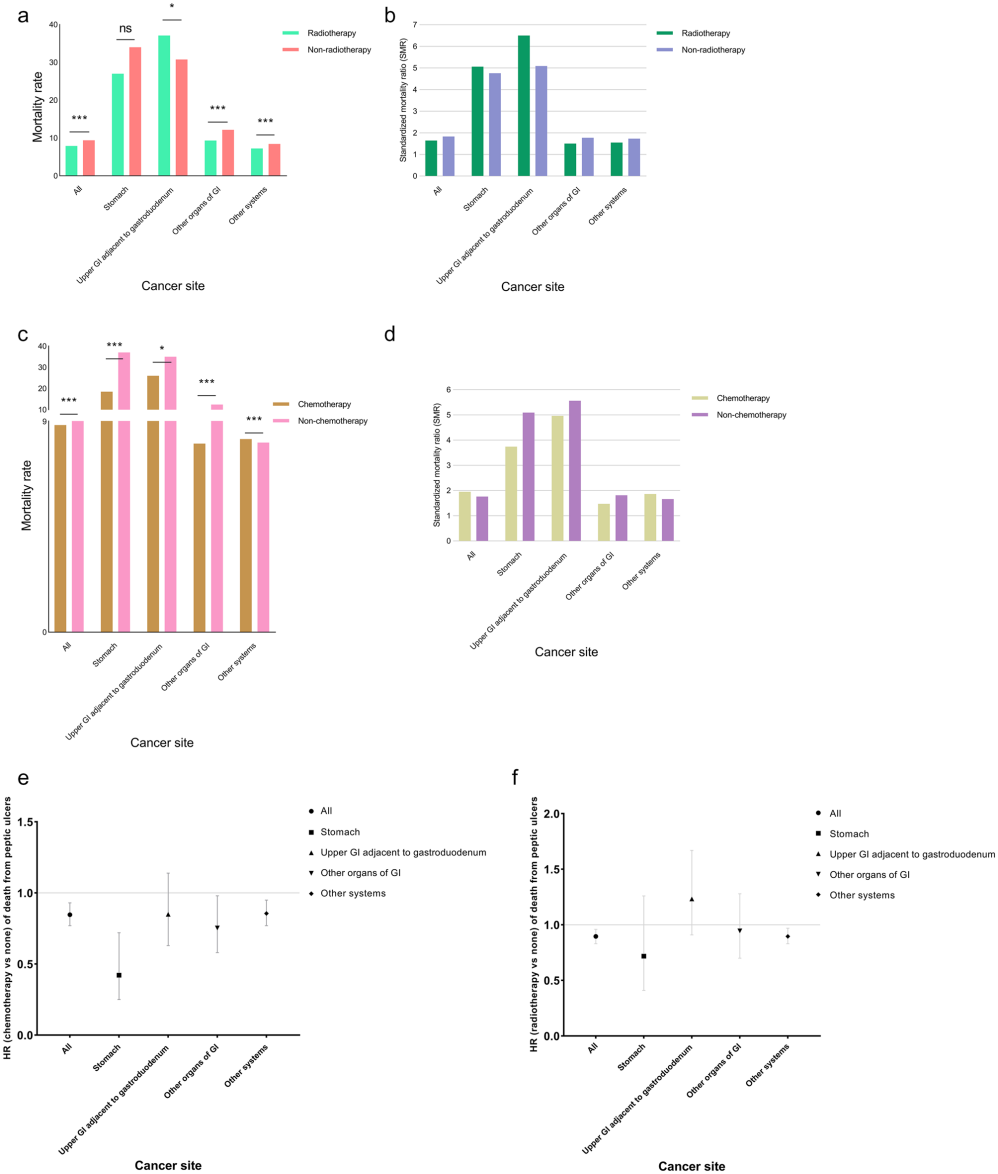
The effect of radio- and chemotherapy on the mortality risk (mortality rate, SMR, and HR) of fatal peptic ulcer by cancer site. The upper gastrointestinal organs (GI) adjacent to gastroduodem refers to the esophagus, small intestine, pancreas, liver, gallbladder, other biliary sites, and other digestive organs. The other organs of the GI include the retroperitoneum, colorectum, and anus. (**a)** Effects of radiotherapy on mortality rate. In patients with upper digestive system cancer adjacent to the gastroduodenum, those receiving radiotherapy were at a higher risk of fatal ulcer than those with no radiation treatment experience. Conversely, patients with other types of cancer treated with radiation had a lower peptic ulcer mortality rate. (**b**) Effects of radiotherapy on SMR. Patients with cancers of the upper GI system treated with radiation had higher relative risks (SMRs) of fatal ulcer than those with no radiotherapy. (**c**) Effects of chemotherapy on mortality rate. A lower peptic ulcer mortality rate was observed among patients with digestive system malignancies who underwent chemotherapy than among those with less chemotherapeutic burden. (**d**) Effects of chemotherapy on SMR. Chemotherapy had a negative effect on the relative risk (SMR) of fatal peptic ulcer in patients with digestive system cancers. (**e**) Effects of radiotherapy on HR (radiotherapy vs none) of peptic ulcer death. In patients with upper digestive system cancer adjacent to the gastroduodenum, those receiving radiotherapy were at higher HR of fatal ulcer than those with no radiation treatment experience. Conversely, radiotherapy reduced the HR of death from peptic ulcers in patients with other types of cancer treated with radiation. (**f**) Effects of chemotherapy on HR (chemotherapy vs none) of peptic ulcer death. For all types of cancer, a lower HR of death from peptic ulcer was observed among patients receiving chemotherapy. *P < 0.05; ***P < 1 × 10^–5^; ns, no statistical significance.

Further analysis indicated that irradiation was a considerable risk factor for fatal peptic ulcers. In patients with upper digestive system cancer adjacent to the gastroduodenum (esophagus, small intestine, pancreas, liver, gallbladder, other biliary sites, and other digestive organs), those who received radiotherapy were at an increased risk relative to those with no radiation treatment (mortality rate, 37.10 vs. 30.79, P < 0.05; hazard ratio [HR] 1.23, 95%CI 0.91-1.67). However, in other types of cancer, patients treated with radiation had a lower risk of peptic ulcer mortality (Figure 4a,e). Consistently, patients with cancers of the upper gastrointestinal system (esophagus, stomach, small intestine, pancreas, liver, gallbladder, other biliary sites, and other digestive organs) who received radiotherapy manifested higher SMRs than those who did not (Figure 4b). In contrast, lower peptic ulcer mortality risk was observed in patients with digestive system malignancies who received chemotherapy compared to those who experienced less chemotherapeutic burden (Figure 4c,f). Chemotherapy had a similar effect on SMRs in patients with upper digestive system cancers (Figure 4d).

### Cancer patients’ risk of fatal peptic ulcer vs. other cancer patients

The aforementioned results (Tables 1, 2, Figs. 1, 2, 3, 4, Supplementary Tables 1-2) illustrate the cancer survivors’ risk of peptic ulcer mortality compared with the general population. Table 3 shows the HRs of the patients who died of peptic ulcer compared with the corresponding patients stratified by subgroup, complementing the above results. The relative risk of fatal peptic ulcer was dramatically higher in the elderly, with the HR of those aged 80+ years vs. ≤ 39 years ng 57.62 (95% CI 42.73–77.70, *P* < 0.001). The relative risk of fatal peptic ulcer was also significantly higher in men than in women (HR = 1.38, 95% CI 1.29–1.47, *P* < 0.001), unmarried vs. married (HR = 1.47, 95% CI 1.38–1.57, *P* < 0.001). Compared to patients with in situ cancer staging, patients with distant cancers had a greater relative risk of peptic ulcer mortality (HR = 2.10, 95% CI 1.75–2.52). When compared to patients diagnosed from 1975–1989, patients diagnosed in more recent years had a lower HR (1990–1999: HR = 0.55; 95% CI 0.51–0.60, *P* < 0.001; 2000–2009: HR = 0.31; 95% CI 0.28–0.33, *P* < 0.001; 2010–2016: HR = 0.25; 95% CI 0.23–0.28, *P* < 0.001). Additional findings included a higher HR for patients who did not receive surgery vs. those undergoing surgery (HR = 1.56, 95% CI 1.45–1.68, *P* < 0.001), non-chemotherapy vs. those receiving chemotherapy (HR = 1.18; 95% CI 1.08–1.30, *P* < 0.001), and non-radiotherapy vs. patients who received radiotherapy (HR = 1.12; 95% CI 1.04–1.20, *P* < 0.01). Moreover, the mortality risk of fatal peptic ulcers varied with cancer sites, with patients having malignancies of the upper digestive system showing significantly greater risk than those with other cancers (other organs of the digestive system vs. upper digestive system: HR = 0.49, 95% CI 0.43–0.56, *P* < 0.001; other systems vs. upper digestive system: HR = 0.47, 95% CI 0.42–0.53, *P* < 0.001).

**Table 3:** Hazard ratios of fatal peptic ulcer among cancer patients.

Characteristic	Hazard ratio	95% CI	P-value
**Age**			
< 39	1.00	–	
40–49	3.31	2.36–4.63	< 0.001
50–59	8.20	6.07–11.09	< 0.001
60–69	14.73	10.95–19.81	< 0.001
70–79	29.32	21.81–39.42	< 0.001
80+	57.62	42.73–77.70	< 0.001
**Sex**			
Female	1.00	–	
Male	1.38	1.29–1.47	< 0.001
**Race**			
White	1.00	–	
Black	1.03	0.92–1.14	0.61
Other	1.21	1.08–1.36	< 0.001
**Marital status**			
Married	1.00	–	
Unmarried	1.47	1.38–1.57	< 0.001
Unknown	0.96	0.84–1.09	0.49
**Stage**			
In situ	1.00	–	
Localized	1.26	1.08–1.47	< 0.01
Reginal	1.92	1.62–2.26	< 0.001
Distant	2.10	1.75–2.52	< 0.001
Unstaged	1.67	1.41–1.97	< 0.001
**Year of diagnosis**			
1975–1989	1.00	–	
1990–1999	0.55	0.51–0.60	< 0.001
2000–2009	0.31	0.28–0.33	< 0.001
2010–2016	0.25	0.23–0.28	< 0.001
**Surgery**			
Yes	1.00	–	
No	1.56	1.45–1.68	< 0.001
Unknown	1.06	0.84–1.35	0.62
**Cancer site**			
Upper digestive system	1.00	–	
Other organs of digestive system	0.49	0.43–0.56	< 0.001
Other systems	0.47	0.42–0.53	< 0.001

## Discussion

Our study revealed an increased risk of fatal peptic ulcer in more than 8 million patients with cancer. The mortality risk of peptic ulcer varied as a function of the follow-up time, cancer site, whether the patient underwent surgery, and other clinical features (age, marital status, and tumor stage). The incidence of peptic ulcer death in cancer survivors was nearly twice that in the general US population. This was the first study to assess the risk of death due to peptic ulcers, independent of the cancer site and treatment approach. Our data demonstrated that peptic ulcer is one of the non-cancer causes of death among cancer survivors and accounts for a certain proportion. As such, it deserves the attention of clinicians, especially oncologists and gastroenterologists.

With improvements in cancer treatments and patient survival, most patients have been dying of non-cancer causes^13^. Although the absolute quantity is unimpressive, peptic ulcer is still one of the notable non-cancer causes of death, and this needs to be taken seriously. The mortality risks were highest in patients with upper digestive system malignancies (esophagus, stomach, small intestine, gallbladder, other biliary, liver, pancreas, and other digestive organs) than in both the general US population and patients with other cancer types throughout the entire follow-up period (Supplementary Table 2, Table 3). The risk of death from peptic ulcer (SMRs and HRs) decreased with the calendar year of diagnosis (Tables 1, 3).

We found that the relative risk in patients receiving surgery was lower than in those who did not undergo surgery (Tables 1, 3). Surgical resection provides the only probability of long-term survival in many types of cancer. However, some tumors, especially upper gastrointestinal malignancies, are insidious with no early symptoms, and few cancer patients have resectable tumors when they seek medical treatment^6,14,15^. In the SEER program, a tumor is possibly unresectable if the patient’s surgery information is recorded as “no.” Radiotherapy and chemotherapy are routine treatments for patients with unresectable tumors. Nevertheless, these treatments are toxic and often damage the gastrointestinal tract, with being peptic ulcers the most common side effect. Radiation could directly damage the extremely radiosensitive columnar epithelial cells located on the mucosal surface, causing mucosal edema and a significant reduction in mucus secretion, followed by effusion and hemorrhage accompanied by the disappearance of granules and cytoplasmic details in the chief and parietal cells^16^. For instance, Chon et al.^15^ found radiation-induced gastric and duodenal ulcers in 32 (26.0%) and 20 (16.3%) patients, respectively, a month after the completion of chemoradiotherapy for advanced hepatocellular carcinoma. In view of our data, we hypothesized that the increased risk of peptic ulcer mortality might be caused by radiation damage to the gastroduodenum during radiotherapy, which could also explain why survivors with upper digestive system malignancies adjacent to the gastroduodenum are more vulnerable to fatal ulcers. Therefore, physicians should be aware that serious ulceration causing lethal hemorrhage or perforation could occur following radiotherapy if the gastroduodenum is within the scope of high-dose radiation. To separate the gastric wall from the high-dose radiation field, it is practical to inject air into the stomach before each irradiation session^18^.

The biological basis underlying the mechanism of gastroduodenal mucosal injury from irradiation has been studied in preclinical studies^6^. Some scholars have approved the presumption of dose-dependent cytotoxicity to stem cells of mucosal crypts with radiation doses above 8 Gy^19^. This results in the progressive injury and denudation of the mucosal wall. Another doctrine holds that irradiation with 15 Gy induces apoptosis of the microvascular endothelium, leading to dysfunction of crypt stem cells and eventually facilitating the failure of crypt regeneration^20^.

When analyzing SMRs by cancer site and follow-up time after diagnosis, we observed a significantly high SMR for the first year after diagnosis, followed by a dramatic decline thereafter. However, for the period of over 10 years following diagnosis, patients with malignancies of the upper gastrointestinal system adjacent to the gastroduodenum showed elevated SMRs for peptic ulcer deaths relative to those with other types of cancer, and these tended to increase over time (Fig. 3). This phenomenon may be due to the acute and long-term toxicities of therapy to the gastroduodenum. The acute effects of therapies may subside, but patients may remain at risk for long-term peptic ulcers and other unexpected gastrointestinal events. For example, an elevated mortality risk of peptic ulcer post-treatment has been observed in patients receiving certain forms of radiotherapy for pancreatic^22^ and esophageal cancer^23^. Similarly, gastroduodenal ulcerations were observed as a delayed complication of hepatic metastasis radioembolization, possibly attributable to radiation-induced damage to the gastroduodenum^4,24,25^.

## Conclusion

Our research showed that the peptic ulcer-specific mortality rate among cancer survivors was nearly twice that of the general US population. The SMRs varied with the age at diagnosis, race, sex, marital status, year of diagnosis, tumor stage, types of cancer, follow-up time, and whether the patients had undergone surgery or radiotherapy. The SMRs were highest in the first year following diagnosis for most tumor types and were significantly higher among patients with upper digestive system malignancies than among those with other types of cancer 10 years after diagnosis. Additionally, both the SMRs and HRs of peptic ulcer mortality were higher in survivors with upper gastrointestinal system malignancies than in those with other cancers. Notably, the elevated risk could be attributed to gastrointestinal toxicity from radiotherapy due to the high radiosensitivity of the gastroduodenum, which deserves attention in the survivorship management of cancer patients. Our findings emphasized the need to develop comprehensive guidelines for gastrointestinal care for targeted subgroups of cancer survivors, which require strengthened cooperation between gastroenterologists and oncologists. Further studies may focus on diminishing the side effects of therapies and on the implementation of appropriate healthcare services to improve the long-term survival of cancer survivors.

## Limitations of the study

We encourage clinical guidelines and survivorship administrations to refer to our analyses in peptic ulcer prevention practice, but our study had limitations. First, the causes of death (including peptic ulcer) may be subject to misclassification bias in the SEER, although such mistakes are uncommon^26,27^. Additionally, patients with a recent diagnosis had limited follow-up time and less opportunity to die from any cause. We attempted to alleviate this restriction by stratifying a more modern timeframe of 2000–2016. Third, despite the advantages of this type of research, the SEER database is far from perfect. It does not include information on the patients’ lifestyle and comorbidities**,** which may aggravate a patient’s risk of ulcer (e.g., aspirin administration for heart disease), or the complete treatment. We acknowledge these restrictions and do not intend to extrapolate our results to specific subgroups beyond the variables investigated in the current research.

## Methods

### Data sources

Patients with malignant cancer diagnosed between 1975 and 2016 were extracted from the Surveillance, Epidemiology, and End Results (SEER) program. SEER is a project that collects data through a network of population-based incident tumor registries, including incidence, survival, and treatment, which cover 28% of US residents^28^. Comorbidities, surgical pathology, margin status, performance status, radiotherapy doses, or chemotherapeutic drugs are not recorded in the SEER database. The SEER 18 registry and client SEER*Stat 8.3.6 were utilized for the present analyses^29^.

### Inclusion criteria and study variables

All patients diagnosed with malignancy were included. Patients diagnosed via autopsy or death certificates were excluded. Leukemia and lymphoma were analyzed as an aggregate group to allow for a more accurate analysis. Data accessible from the SEER registry include the age at diagnosis, sex, race, year of diagnosis, marital status, anatomic site of disease, stage of cancer, and vital status at the last follow-up. Survival duration after diagnosis was measured in months in SEER, with the minimal value for any event equal to one month. Patients with a survival time of less than a full month following diagnosis were recorded as zero in the SEER. According to standard epidemiologic conventions, these patients were assigned a survival duration of half a month for the current analyses^30^. Patients were considered to have died of peptic ulcer if the cause of death variable was stated as “stomach and duodenal ulcers” (ICD-10 code K25-K26).

### Statistical analyses

Ulcer mortality (number of deaths from stomach and duodenal ulcers divided by person-years of survival) was calculated to estimate the prevalence of fatal ulcers in the subgroups of cancer survivors. Analyses of the relationship between peptic ulcer death and demographic characteristics were adjusted for the age (at diagnosis), sex, and race distribution of the patients in the SEER program. The stage of prostate cancer, recorded as locoregional in SEER, was classified as the localized subgroup. The standardized mortality ratios (SMRs) and 95% CIs, which represent the relative risk of death for survivors with cancer compared with the general US population, were stratified by cancer subgroup and calculated as previously described^30–32^. The relative risk of fatal peptic ulcer over the follow-up time after diagnosis was characterized by the SMR that was standardized by age (at diagnosis), race and sex distribution of cancer patients. To compare cancer patients’ risk of fatal peptic ulcer with other cancer patients, we performed a survival analysis using a Cox proportional hazards model to calculate the hazard ratios (HRs), with the survival time from diagnosis until fatal peptic ulcer and non-ulcer deaths plus living patients being censored. Statistical analyses were conducted using STATA version 15.0 (StataCorp, Texas, USA; https://www.stata.com/) and Microsoft Excel 2013 (Microsoft, Redmond, WA, USA; https://www.microsoft.com/).

## Supplementary Information

Supplementary Information 1.

Supplementary Information 2.

## Data Availability

The data are provided in the SEER database, which is freely accessible to the public. The source data underlying all tables and figures were freely available in the SEER database.
