# Supplementary material for: Incidence and characteristics of death from peptic ulcer among cancer patients in the United States
Source: Sci Rep. 2021 Dec 8;11:23579. doi: 10.1038/s41598-021-00602-1 (PMC8654846; doi:10.1038/s41598-021-00602-1)
Supplement: Supplementary file 1 — Supplementary Information 1. [file 41598_2021_602_MOESM1_ESM.docx]

| Supplementary table 1. Gastroduodenal ulcer mortality risk in patients with cancer by site and years since diagnosis | | |  |  |  |  |  |  |  |  |  |  |  |  |
| --- | --- | --- | --- | --- | --- | --- | --- | --- | --- | --- | --- | --- | --- | --- |
| cancer site | | |  |  |  |  |  |  |  |  |  |  |  |  |
|  |  |  | ＜1 | | | 1-5 | | | 5-10 | | | ＞10 | | |
|  |  |  | No.of deaths | SMR^†‡^ | 95%CI | No.of deaths | SMR^†‡^ | 95%CI | No.of deaths | SMR^†‡^ | 95%CI | No.of deaths | SMR^†‡^ | 95%CI |
| All | | | 1426 | 10.23 | 9.94-10.53 | 1348 | 2.77 | 2.68-2.87 | 945 | 2.76 | 2.64-2.89 | 979 | 2.55 | 2.39-2.71 |
| digestive system | Stomach | | 86 | 16.66 | 13.96-19.87 | 19 | 3.34 | 2.42-4.61 | 12 | 3.64 | 2.30-5.78 | 6 | 2.49 | 1.12-5.54 |
|  | upper GI adjacent to Gastroduodenum | Other Digestive organs | 5 | 20.58 | 8.56-49.44 | 0 | 0.00 | NA | 0 | 0.00 | NA | 0 | 0.00 | NA |
|  |  | Pancreas | 69 | 12.93 | 10.53-15.88 | 16 | 5.13 | 3.38-7.78 | 5 | 5.61 | 2.52-12.50 | 1 | 2.69 | 0.38-19.12 |
|  |  | Liver | 47 | 15.80 | 12.24-20.39 | 10 | 3.06 | 1.74-5.38 | 1 | 2.16 | 0.54-8.64 | 1 | 4.18 | 0.59-29.66 |
|  |  | Other Biliary | 11 | 13.69 | 8.93-21.00 | 6 | 5.94 | 3.20-11.04 | 0 | 0.00 | NA | 4 | 15.24 | 5.72-40.61 |
|  |  | Gallbladder | 4 | 13.46 | 7.97-22.73 | 9 | 7.47 | 4.02-13.89 | 1 | 1.82 | 0.26-12.92 | 0 | 0.00 | NA |
|  |  | Small Intestine | 16 | 18.32 | 12.56-26.72 | 5 | 3.11 | 1.72-5.62 | 2 | 3.43 | 1.54-7.64 | 4 | 5.34 | 2.00-14.23 |
|  |  | Esophagus | 13 | 6.56 | 4.40-9.79 | 9 | 2.68 | 1.49-4.85 | 1 | 1.37 | 0.34-5.49 | 1 | 1.85 | 0.26-13.14 |
|  | other organs of GI | Retroperitoneum | 1 | 16.17 | 6.07-43.08 | 1 | 5.79 | 1.87-17.97 | 1 | 7.98 | 1.99-31.89 | 1 | 7.88 | 1.11 -55.97 |
|  |  | Anus | 5 | 11.73 | 7.19-19.15 | 4 | 3.23 | 1.79-5.83 | 4 | 3.86 | 1.84-8.10 | 3 | 3.29 | 1.06-10.20 |
|  |  | Colorectum | 150 | 10.43 | 9.64-11.29 | 160 | 3.01 | 2.75-3.29 | 149 | 3.37 | 3.01-3.77 | 159 | 2.97 | 2.55-3.47 |
| other systems | | Cervix Uteri§ | 7 | 28.58 | 21.19-38.53 | 13 | 10.32 | 7.44-14.30 | 8 | 10.19 | 6.77-15.34 | 15 | 7.40 | 4.46-12.27 |
|  |  | Lung | 279 | 10.80 | 9.86-11.82 | 113 | 3.71 | 3.22-4.27 | 54 | 4.42 | 3.55-5.51 | 26 | 3.59 | 2.44-5.27 |
|  |  | Trchea, Mediastinum and Other Respiratory Organs | 1 | 14.58 | 2.05-103.52 | 0 | 0.00 | NA | 0 | 0.00 | NA | 0 | 0.00 | NA |
|  |  | Bones | 2 | 12.43 | 4.67-33.12 | 0 | 0.00 | NA | 1 | 4.97 | 1.24-19.88 | 1 | 3.49 | 0.49-24.79 |
|  |  | Brain | 14 | 7.96 | 5.14-12.34 | 4 | 2.66 | 1.19-5.92 | 1 | 2.40 | 0.60-9.61 | 1 | 3.00 | 0.42-21.28 |
|  |  | Breast^§^ | 82 | 11.77 | 10.89-12.72 | 186 | 3.26 | 3.00-3.54 | 167 | 3.34 | 3.01-3.69 | 201 | 2.93 | 2.55-3.36 |
|  |  | Uterus^§^ | 19 | 19.52 | 16.88-22.59 | 45 | 6.08 | 5.21-7.10 | 39 | 6.37 | 5.32-7.64 | 78 | 4.95 | 3.97-6.18 |
|  |  | Other Nervous System | 15 | 4.32 | 3.00-6.21 | 11 | 0.81 | 0.48-1.37 | 2 | 0.44 | 0.14-1.36 | 1 | 1.30 | 0.18-9.22 |
|  |  | Endocrine System | 7 | 6.58 | 4.68-9.26 | 11 | 1.73 | 1.18-2.54 | 6 | 1.69 | 1.02-2.81 | 9 | 1.93 | 1.00-3.71 |
|  |  | Eye and Orbit | 1 | 15.90 | 9.59-26.38 | 6 | 5.14 | 3.04-8.67 | 2 | 5.23 | 2.62-10.46 | 6 | 6.90 | 3.10-15.35 |
|  |  | Kidney and Renal Pelvis | 37 | 11.47 | 9.64-13.64 | 34 | 3.15 | 2.56-3.87 | 30 | 3.56 | 2.75-4.61 | 27 | 3.55 | 2.43-5.17 |
|  |  | Larynx | 16 | 16.95 | 13.43-21.39 | 24 | 4.82 | 3.70-6.27 | 17 | 4.58 | 3.22-6.52 | 14 | 3.35 | 1.99-5.66 |
|  |  | Leukemia | 40 | 9.65 | 7.97-11.67 | 40 | 2.68 | 2.10-3.41 | 16 | 2.29 | 1.56-3.37 | 10 | 2.18 | 1.17-4.06 |
|  |  | Lymphoma | 111 | 11.07 | 9.62-12.73 | 33 | 1.95 | 1.58-2.41 | 27 | 2.26 | 1.72-2.96 | 25 | 2.40 | 1.62-3.56 |
|  |  | Miscellaneous | 92 | 11.51 | 9.80-13.51 | 38 | 2.59 | 1.99-3.35 | 12 | 2.40 | 1.53-3.77 | 7 | 3.40 | 1.62-7.14 |
|  |  | Myeloma | 33 | 9.76 | 7.61-12.51 | 20 | 2.34 | 1.62-3.36 | 8 | 2.50 | 1.30-4.80 | 1 | 1.13 | 0.16-8.04 |
|  |  | Oral Cavity and Pharynx | 50 | 16.64 | 14.30-19.37 | 58 | 4.98 | 4.16-5.97 | 26 | 4.71 | 3.65-6.07 | 33 | 4.79 | 3.40-6.73 |
|  |  | Ovary^§^ | 10 | 8.43 | 6.02-11.80 | 7 | 3.08 | 2.07-4.60 | 8 | 4.64 | 2.88-7.46 | 9 | 3.35 | 1.74-6.43 |
|  |  | Prostate^§^ | 107 | 7.93 | 7.40-8.49 | 276 | 2.09 | 1.94-2.25 | 228 | 1.85 | 1.68-2.03 | 204 | 1.60 | 1.40-1.84j6 |
|  |  | Skin non Basal and Squamous | 17 | 5.37 | 4.58-6.29 | 48 | 1.62 | 1.37-1.92 | 39 | 1.80 | 1.46-2.22 | 49 | 1.86 | 1.40-2.46 |
|  |  | Soft Tissue | 2 | 6.02 | 3.49-10.36 | 5 | 2.15 | 1.19-3.89 | 3 | 2.22 | 1.00-4.93 | 3 | 1.87 | 0.60-5.80 |
|  |  | Testis^§^ | 0 | 0.00 | NA | 2 | 5.38 | 2.42-11.97 | 1 | 4.34 | 1.63-11.56 | 3 | 2.86 | 0.92-8.86 |
|  |  | Bladder | 58 | 9.08 | 8.06-10.23 | 103 | 2.64 | 2.31-3.02 | 53 | 2.42 | 2.00-2.92 | 56 | 2.37 | 1.83-3.08 |
|  |  | Vulva^§^ | 9 | 21.10 | 15.41-28.88 | 9 | 6.07 | 4.25-8.69 | 10 | 7.19 | 4.68-11.02 | 11 | 6.46 | 3.58-11.67 |
|  |  | Kaposi Sarcoma | 1 | 19.33 | 10.06-37.14 | 3 | 6.33 | 3.17-12.66 | 3 | 6.90 | 2.87-16.57 | 2 | 4.88 | 1.22-19.50 |
|  |  | Mesothelioma | 2 | 4.23 | 1.59-11.28 | 2 | 2.97 | 0.74-11.89 | 0 | 0.00 | NA | 0 | 0.00 | NA |
|  |  | Nose, Nasal Cavity and Middle Ear | 2 | 10.67 | 5.09-22.38 | 2 | 3.50 | 1.46-8.40 | 3 | 4.24 | 1.37-13.16 | 0 | 0.00 | NA |
|  |  | Other Female Genital Organs§ | 0 | 0.00 | NA | 1 | 6.98 | 2.62-18.61 | 2 | 11.67 | 3.76-36.18 | 1 | 6.26 | 0.88-44.46 |
|  |  | Other Mmale Genital Organs§ | 0 | 0.00 | NA | 2 | 6.07 | 1.96-18.81 | 0 | 0.00 | NA | 1 | 5.77 | 0.81-40.96 |
|  |  | Other Urinary Organs | 1 | 7.21 | 2.33-22.35 | 2 | 2.15 | 0.54-8.60 | 0 | 0.00 | NA | 0 | 0.00 | NA |
|  |  | Penis§ | 2 | 12.15 | 6.32-23.35 | 3 | 3.52 | 1.68-7.38 | 2 | 3.49 | 1.31-9.30 | 2 | 3.00 | 0.75-12.00 |
|  |  | Peritoneum, Omentum and Mesentery | 0 | 0.00 | NA | 1 | 1.59 | 0.22-11.29 | 0 | 0.00 | NA | 0 | 0.00 | NA |
|  |  | Pleura | 0 | 0.00 | NA | 0 | 0.00 | NA | 0 | 0.00 | NA | 0 | 0.00 | NA |
|  |  | Ureter | 1 | 9.39 | 4.69-18.77 | 4 | 3.44 | 1.64-7.23 | 1 | 2.99 | 0.97-9.28 | 2 | 4.06 | 1.01-16.23 |
|  |  | Vagina§ | 1 | 12.67 | 5.27-30.45 | 3 | 4.43 | 1.66-11.79 | 0 | 0.00 | NA | 1 | 3.13 | 0.44 -22.22 |

†Adjusted for the age and sex distributions of patients.

‡Reference population: general US population, 1969 to 2016.

§Sex-specific analysis, adjusted for the age distribution of patients.
