# Supplementary material for: Incidence and characteristics of death from peptic ulcer among cancer patients in the United States
Source: Sci Rep. 2021 Dec 8;11:23579. doi: 10.1038/s41598-021-00602-1 (PMC8654846; doi:10.1038/s41598-021-00602-1)
Supplement: Supplementary file 2 — Supplementary Information 2. [file 41598_2021_602_MOESM2_ESM.docx]

| Supplementary table 2. Mortality rates of fatal gastroduodenal ulcer by anatomic site of cancer | | | |  |  | |  |  |  |  |  |  |  |  |  |  |
| --- | --- | --- | --- | --- | --- | --- | --- | --- | --- | --- | --- | --- | --- | --- | --- | --- |
| cancer site | | | No. of deaths | No of patients | | person-years | | male and female | | | female¶ | | | male¶ | | |
|  |  |  |  |  |  |  |  | mortality risk†‡ | SMR‡§ | 95% CI‡§ | mortality risk†‡ | SMR‡§ | 95% CI‡§ | mortality risk†‡ | SMR‡§ | 95% CI‡§ |
| digestive system | Stomach | | 123 | 138,776 | | 362179 | | 25.17 | 4.76 | 3.99-5.68 | 22.18 | 6.12 | 4.71-7.96 | 28.73 | 4.02 | 3.16-5.11 |
|  | upper GI adjacent to Gastrod-uodenum | Other Digestive Organs | 5 | 8,278 | | 7921 | | 66.79 | 9.84 | 4.09-23.64 | 15.27 | 4.09 | 0.58-29.04 | 128.09 | 15.16 | 5.69-40.41 |
|  |  | Pancreas | 91 | 195,663 | | 184030 | | 40.63 | 7.13 | 5.8-8.75 | 33.16 | 7.61 | 5.64-10.26 | 49.52 | 6.74 | 5.08-8.95 |
|  |  | Liver | 59 | 118,324 | | 178461 | | 30.05 | 6.69 | 5.18-8.63 | 18.65 | 6.50 | 3.85-10.97 | 43.63 | 6.75 | 5.04-9.04 |
|  |  | Other Biliary | 21 | 28,567 | | 54453 | | 29.71 | 5.21 | 3.4-8.00 | 12.63 | 3.20 | 1.33-7.69 | 50.03 | 6.49 | 3.98-10.59 |
|  |  | Gallbladder | 14 | 22,340 | | 43804 | | 23.19 | 4.40 | 2.61-7.43 | 25.75 | 5.66 | 3.21-9.97 | 20.14 | 1.88 | 0.47-7.53 |
|  |  | Small Intestine | 27 | 29,300 | | 144654 | | 20.25 | 3.60 | 2.47-5.25 | 14.72 | 3.38 | 1.82-6.28 | 26.82 | 3.74 | 2.33-6.02 |
|  |  | Esophagus | 24 | 73,170 | | 136295 | | 15.61 | 2.46 | 1.65-3.67 | 13.61 | 2.58 | 1.07-6.2 | 17.99 | 2.43 | 1.55-3.81 |
|  | other organs of GI | Retroperitoneum | 4 | 7,011 | | 37305 | | 12.33 | 3.50 | 1.31-9.33 | 6.11 | 2.02 | 0.28-14.36 | 19.73 | 4.63 | 1.49-14.35 |
|  |  | Anus | 16 | 34,322 | | 205924 | | 10.13 | 2.13 | 1.31-3.48 | 8.51 | 2.49 | 1.34-4.62 | 12.06 | 1.73 | 0.78-3.84 |
|  |  | Colon and Rectum | 618 | 836,720 | | 5080314 | | 9.16 | 1.72 | 1.59-1.86 | 7.42 | 1.91 | 1.7-2.14 | 11.23 | 1.57 | 1.41-1.75 |
| other systems | | Cervix Uteri | 43 | 87,411 | | 766854 | | 6.19 | 4.63 | 3.44-6.25 | 11.40 | 4.63 | 3.44-6.25 | 0.00 | 0.00 | NA |
|  |  | Lung and Bronchus | 472 | 965,341 | | 1812077 | | 21.12 | 3.90 | 3.56-4.27 | 12.01 | 3.18 | 2.7-3.74 | 31.97 | 4.34 | 3.89-4.84 |
|  |  | Trachea, Mediastinum and Other Respiratory Organs | 1 | 3,621 | | 22078 | | 26.33 | 3.73 | 0.53-26.5 | 48.46 | 13.15 | 1.85-93.35 | 0.00 | 0.00 | 0-0 |
|  |  | Vulva | 39 | 48,635 | | 437415 | | 6.98 | 3.42 | 2.5-4.68 | 12.84 | 3.42 | 2.5-4.68 | 0.00 | 0.00 | NA |
|  |  | Brain | 20 | 123,661 | | 459463 | | 15.54 | 3.37 | 2.17-5.22 | 14.08 | 3.78 | 1.89-7.56 | 17.27 | 3.14 | 1.78-5.53 |
|  |  | Miscellaneous | 149 | 243,385 | | 576313 | | 20.18 | 3.31 | 2.82-3.89 | 13.88 | 3.12 | 2.42-4.03 | 27.67 | 3.45 | 2.81-4.25 |
|  |  | Oral Cavity and Pharynx | 167 | 194,033 | | 1125688 | | 16.94 | 3.15 | 2.71-3.67 | 15.17 | 4.19 | 3.24-5.43 | 19.05 | 2.79 | 2.31-3.36 |
|  |  | Other Female Genital Organs | 4 | 8,685 | | 51479 | | 5.35 | 3.15 | 1.18-8.39 | 9.85 | 3.15 | 1.18-8.39 | 0.00 | 0.00 | NA |
|  |  | Kaposi Sarcoma | 9 | 22,244 | | 98175 | | 223.20 | 3.14 | 1.64-6.04 | 393.10 | 3.32 | 0.83-13.28 | 21.03 | 3.09 | 1.48-6.49 |
|  |  | Larynx | 71 | 69,152 | | 452396 | | 14.24 | 2.67 | 2.12-3.37 | 11.70 | 4.92 | 2.92-8.31 | 17.26 | 2.40 | 1.85-3.12 |
|  |  | Myeloma | 62 | 101,626 | | 351116 | | 13.22 | 2.67 | 2.08-3.42 | 10.96 | 3.36 | 2.33-4.83 | 15.92 | 2.26 | 1.6-3.17 |
|  |  | Other Male Genital Organs | 3 | 2,185 | | 17296 | | 9.32 | 2.64 | 0.85-8.19 | 0.00 | 0.00 | NA | 20.42 | 2.64 | 0.85-8.19 |
|  |  | Uterus | 181 | 250,987 | | 2179474 | | 4.67 | 2.59 | 2.23-2.99 | 8.59 | 2.59 | 2.23-2.99 | 0.00 | 0.00 | NA |
|  |  | Eye and Orbit | 15 | 16,780 | | 141603 | | 17.25 | 2.47 | 1.49-4.10 | 13.49 | 4.09 | 2.05-8.18 | 21.72 | 1.70 | 0.81-3.57 |
|  |  | Vagina | 5 | 10,244 | | 76860 | | 4.14 | 2.33 | 0.97-5.60 | 7.62 | 2.33 | 0.97-5.6 | 0.00 | 0.00 | NA |
|  |  | Bones | 4 | 16,674 | | 129231 | | 12.58 | 2.32 | 0.87-6.19 | 6.42 | 1.55 | 0.22-10.99 | 19.91 | 2.79 | 0.9-8.64 |
|  |  | Mesothelioma | 4 | 15,597 | | 22166 | | 8.61 | 2.25 | 0.84-5.99 | 0.00 | 0.00 | NA | 18.86 | 2.79 | 1.05-7.43 |
|  |  | Nose, Nasal Cavity and Middle Ear | 7 | 12,018 | | 65951 | | 10.70 | 2.21 | 1.06-4.64 | 9.12 | 2.75 | 0.89-8.54 | 12.57 | 1.93 | 0.72-5.14 |
|  |  | Lymphoma | 196 | 365,017 | | 2329566 | | 10.22 | 2.07 | 1.8-2.38 | 8.76 | 2.41 | 1.97-2.95 | 11.97 | 1.83 | 1.51-2.22 |
|  |  | Leukemia | 106 | 219,261 | | 1079239 | | 10.86 | 2.06 | 1.7-2.49 | 7.42 | 1.93 | 1.4-2.68 | 14.95 | 2.12 | 1.68-2.69 |
|  |  | Kidney and Renal Pelvis | 128 | 219,761 | | 1257816 | | 10.43 | 2.01 | 1.69-2.39 | 8.69 | 2.44 | 1.84-3.25 | 12.50 | 1.82 | 1.47-2.27 |
|  |  | Penis | 9 | 9,765 | | 71567 | | 5.69 | 1.98 | 1.03-3.81 | 0.00 | 0.00 | NA | 12.46 | 1.98 | 1.03-3.81 |
|  |  | Ovary | 34 | 131,819 | | 754837 | | 3.38 | 1.87 | 1.34-2.62 | 6.23 | 1.87 | 1.34-2.62 | 0.00 | 0.00 | NA |
|  |  | Ureter | 8 | 9,154 | | 49262 | | 8.14 | 1.83 | 0.91-3.65 | 0.94 | 0.70 | 0.1-5 | 16.71 | 2.36 | 1.13-4.96 |
|  |  | Testis | 6 | 53,702 | | 630100 | | 3.36 | 1.76 | 0.79-3.91 | 0.00 | 0.00 | NA | 7.36 | 1.76 | 0.79-3.91 |
|  |  | Breast | 636 | 1,385,735 | | 12000000 | | 5.02 | 1.58 | 1.46-1.71 | 5.56 | 1.59 | 1.47-1.72 | 4.37 | 0.63 | 0.2-1.94 |
|  |  | Urinary Bladder | 270 | 310,644 | | 2115298 | | 9.51 | 1.51 | 1.34-1.7 | 8.60 | 1.98 | 1.56-2.52 | 10.59 | 1.40 | 1.22-1.61 |
|  |  | Other Urinary Organs | 3 | 4,907 | | 26116 | | 18.77 | 1.47 | 0.47-4.56 | 29.96 | 4.42 | 1.11-17.68 | 5.45 | 0.63 | 0.09-4.48 |
|  |  | Soft Tissue including Heart | 13 | 51,819 | | 350730 | | 6.47 | 1.12 | 0.65-1.93 | 4.76 | 1.23 | 0.51-2.95 | 8.49 | 1.06 | 0.53-2.13 |
|  |  | Prostate | 815 | 1,153,981 | | 9095242 | | 3.33 | 1.02 | 0.95-1.09 | 0.00 | 0.00 | NA | 7.30 | 1.02 | 0.95-1.09 |
|  |  | Endocrine System | 33 | 243,115 | | 1955341 | | 5.21 | 0.98 | 0.7-1.38 | 2.59 | 0.74 | 0.43-1.28 | 8.32 | 1.24 | 0.8-1.93 |
|  |  | Other Nervous System | 29 | 124,837 | | 617984 | | 4.44 | 0.92 | 0.64-1.32 | 3.01 | 0.96 | 0.61-1.51 | 6.14 | 0.84 | 0.45-1.56 |
|  |  | Skin non Basal and Squamous | 153 | 494,208 | | 4146605 | | 4.41 | 0.82 | 0.7-0.96 | 3.80 | 1.09 | 0.85-1.39 | 5.14 | 0.70 | 0.57-0.86 |
|  |  | Peritoneum, Omentum and Mesentery | 1 | 8,095 | | 27681 | | 22.70 | 0.81 | 0.11-5.73 | 0.00 | 0.00 | NA | 49.71 | 7.18 | 1.01-50.96 |
|  |  | Pleura | 0 | 481 | | 1215 | | 0.00 | 0.00 | NA | 0.00 | 0.00 | NA | 0.00 | 0.00 | NA |

†Per 100,000 person-years.

‡Adjusted for age, race, and sex distributions of patients.

§Reference population: general US population, 1969 to 2016.

¶Sex-specific analysis, adjusted for age and race distributions of patients.
